# Early Evidence of Parental Attachment Among Polish Adolescents

**DOI:** 10.5964/ejop.v16i1.1809

**Published:** 2020-03-03

**Authors:** Elisa Delvecchio, Jian-Bin Li, Hanna Liberska, Adriana Lis, Claudia Mazzeschi

**Affiliations:** a Department of Philosophy, Social Sciences and Education, University of Perugia, Perugia, Italy; b Department of Early Childhood Education, The Education University of Hong Kong, Hong Kong; c Department of Social Psychology and Research on Youth, Kazimierz Wielki University, Bydgoszcz, Poland; d Department of Developmental Psychology and Socialization, University of Padova, Padova, Italy; The Maria Grzegorzewska University Warsaw, Poland

**Keywords:** attachment, adolescence, self-esteem, Poland, cross-cultural study

## Abstract

Parental attachment is important for adolescents’ development as well as cultural context. Poland used to be collectivist but now is closer to individualistic due to social and economic transformation. Few studies have examined parental attachment and self-esteem among Polish adolescents. This descriptive study (N = 303 Polish adolescents) investigated the levels of parental attachment, gender differences, preferred attachment figure, association with self-esteem and cultural differences with collectivistic (China) and individualistic (Italy) cultures. The results indicated that: (1) there was no gender difference in parental attachment; (2) mother was the preferred attachment figure; (3) parental attachment was related to self-esteem; and (4) cultural differences were found. Findings were discussed in terms of Polish sociopolitical situation.

Parental attachment refers to an affectional bond with parents. Although usually attachment to parents develops in infancy (Bowlby, 1969), it continues to affect the individual’s psychological adjustment during adolescence (Cai, Hardy, Olsen, Nelson, & Yamawaki, 2013; Tambelli, Laghi, Odorisio, & Notari, 2012). This makes parents remain a fundamental source of emotional support for their adolescent children even though they become increasingly independent and autonomous (Buist, Dekovic, Meeus, & van Aken, 2002; Cai et al., 2013).

Prior research has found that parental attachment varies across cultural values and family orientation (Chen, French, & Schneider, 2006; Li, Delvecchio, Miconi, Salcuni, & Di Riso, 2014; Song, Thompson, & Ferrer, 2009; Trommsdorff, 2006), suggesting that cultural context should be taken into account in order to better understand attachment to parents. Poland is a country with specific cultural context. Although prior research has addressed Polish young adults’ attachment in a retrospective study (Hardt, Dragan, Schultz, & Engfer, 2011), little has been done to study parental attachment among Polish adolescents. The current research aimed to address this void using a widely used self-report measure specifically devised for the assessment of parental attachment among adolescents: the revised version of the Inventory of Parent and Peer Attachment (IPPA-R; Armsden & Greenberg, 2001).

## Brief Introduction of the Cultural Changes in Poland

As highlighted by Stefaniak and Bilewicz (2016), the Polish host several ethical, religious and social differences in one culture, due to their historical issue (i.e., the World War II) and the fast economic development. Poland is a country where the collapse of communism and its collectivistic culture twenty years ago triggered many changes at the economic, social and individual level. Nowadays, Poland is categorized as an individualistic country, as indicated by a score of 60/100 on individualism (https://geert-hofstede.com/poland.html). In sum, Poland used to be a collectivistic country but now is closer to be individualistic due to the sociopolitical transformation it has undergone. Social norms of behavior, working rules, values, parental and gender roles that successfully organized the life of individuals and their families for the last fifty years during the collectivistic communist culture became non-adaptive and useless. This phenomenon, defined in terms of social anomy, must have had an impact on the family processes, especially with regard to the family orientation which is considered to be the core for Polish culture (Boski, 2009; Inglehart & Welzel, 2005; Nowak, 1979; Siemieńska, 2008). Social anomy may lead to differences in attachment. There is scarce literature about parental attachment among adolescents in Poland. The current study aimed to examine four issues described below.

## Gender Differences in Attachment

Gender differences in levels of attachment were examined. This is a controversial topic in the literature because of inconsistent findings (Buist et al., 2002; Delvecchio, 2013; Kenny & Rice, 1995, Li et al., 2014; Li, Delvecchio, Lis, Nie, & Di Riso, 2015; San Martini, Zavattini, & Ronconi, 2009; Song et al., 2009). As example, Rice, Cunningham, and Young (1997), referring to the “allegiance” effect proposed that maternal relationships were more influential for girls and paternal relationships were more influential for boys. However, more recently Li, Lis, and Delvecchio (2016) found girls reporting stronger maternal attachment than boys but no differences according to fathers’ attachment. In light of the cultural changes concerning gender roles occurred in Poland, it was important to understand whether girls’ parental attachment was stronger than in boys. However, because there were no empirical studies with the IPPA-R on possible gender differences in attachment scores in the Polish, no specific hypotheses were made.

## Preferred Attachment Figure

Although fathers in Poland formally maintained the position of the head of the family. Poland is characterized by a domestic matriarchy: the family is ruled by the mother rather than the father, and mothers in Poland are also primary caregiver and more involved than fathers in child-rearing process (Walczewska, 1999). In light of this, we hypothesized that adolescents would report higher maternal attachment than paternal attachment.

## Association With Self-Evaluation

Attachment theory assumes that children in secure and supportive parent-child relationships are more likely to perceive themselves positively and more competent compared with children in insecure or unsupportive relationships (see Cassidy, 2008; Weinfield, Sroufe, Egeland, & Carlson, 2008). Attachment theorists have also recognized that continued parental support is required to maintain positive self-regard as children mature (Thompson, 2008). Many studies supported empirically this view finding a significant association of adolescent attachment to mothers and fathers with self-esteem and self-appraisal (Armsden & Greenberg, 1987; Cotterell, 1992; Laible, Carlo, & Roesch, 2004; Noom, Deković, & Meeus, 1999; O’Koon, 1997; Song et al., 2009; Wilkinson, 2004). In summary, the existing literature has shown that relationships with both parents are associated with self-evaluation among adolescents, but little is known about whether this result could be replicated among Polish adolescents.

## Cultural Differences

The current study also compared Polish adolescents’ attachment scores with two other countries where the meaning of family and family bonding are partly similar and partly different: China (collectivistic), Italy (individualistic). Parent-adolescent relationships have been shown to vary across cultural values and family orientation, which may lead to differences in parental attachment (Chen, French, & Schneider, 2006; Li et al., 2014; Song et al., 2009; Trommsdorff, 2006). Few research has been carried out comparing attachment levels in adolescents from different countries. As example, Li et al. (2014) compared parental attachment among Chinese and Italian adolescents showing that parental attachment in Chinese adolescents was lower than their Italian counterparts. The authors suggested that the influence of filial piety and Confucian beliefs which emphasize the importance of parental control and lower expression of love and emotions, may affect Chinese relationships showing higher emotional distance between parents and child. Moreover, Chinese parents more often refer to authoritative parenting style, discouraging adolescents to get closer for emotional support. On the other hand, although also in Italy a family orientation is present, parent-child relationships are characterized by a bidirectional path through which autonomy, support, emotional bonding and reciprocal respect are fundamental. Hardt et al. (2011) comparing parent-child relationships in Poland and Germany found that Polish mothers and fathers were evaluated as stronger and weaker, respectively, than German ones. The authors suggested that a possible explanation may refer to Polish domestic matriarchy, which see the family ruled by the mother than the father although the father maintained his position as the head of the family (Walczewska, 1999). However, it is important to underline that those data were collected retrospectively (i.e., they captured mainly the post World-War II era) thus they may not be truly representative of the current situation.

To the best of our knowledge, no more recent comparison studies in adolescent samples involving the Polish were available. Thus, differences in attachment scores in Polish adolescents were left as an open question due to insufficient data.

## The Current Study

The present study was designed as an initial investigation of attachment among Polish adolescents, an understudied population in the literature of the field, by examining levels of attachment to mothers and fathers, analyzing gender differences and the preferred figure of attachment, associating it with social development correlates (i.e., self-esteem), and comparing it with previous cross-cultural findings. We hoped that the findings of this study would contribute to our knowledge of adolescent attachment in the current cultural context of Poland, a country that has undergone changes in values from collectivism to individualism.

## Method

### Participants

A total of 303 Polish (176 boys, 127 girls; *M*_age_ = 16.86 years, *SD* = 0.87 years adolescents) were recruited from middle and high schools (Grade 7 to 11) in Bydgoszcz (Poland). Families came from a working and middle-class background. Involved families were required to indicate whether they were immigrants and only genuine Polish families were selected in order to avoid language and culture-based problems. Approximately 94% of the families who received the leaflet agreed to participate. Those who declined due to lack of interest and concerns about sharing personal information. Exclusion criteria included psychiatric hospitalization, psychological treatment or testing within the past 2 years.

### Measures

#### Parental Attachment

The revised version of the Inventory of Parent and Peer Attachment (IPPA-R; Armsden & Greenberg, 1987) was employed to assess adolescents’ cognitive perception and feelings towards mother and father on a 5-point Likert scale (from “1 = never” to “5 = always”). This scale consists of 25 items measuring the extent of three dimensions (i.e., *Trust*, *Communication*, and *Alienation*) separately to each of the attachment figures with parallel wordings of items. IPPA-R yields a total attachment score based on the sum of item ratings with *Alienation* reverse scored. Higher scores indicate stronger attachment. Sample items are “I am angry at my mother” (reverse score) and “my mother respects my feelings”. This instrument has been extensively used in the literature, showing overall adequate psychometric characteristics. Comparatively much less studies have used this scale in Polish samples (Farnicka & Grzegorzewska, 2015; Gajewski & Małkowska-Szkutnik, 2012; Grzegorzewska, 2013; Mazur & Małkowska-Szkutnik, 2011). Polish adolescents answered the existing Polish version of this measure (Farnicka & Grzegorzewska, 2015). In the current study, Cronbach’s α was .95 for total paternal attachment, .90 for paternal trust, .90 for paternal communication, .80 for paternal alienation, .95 for total maternal attachment, .92 for maternal trust, .88 for maternal communication, and .85 for maternal alienation.

#### Self-Esteem

The Rosenberg Self-Esteem Scale (RSES; Rosenberg, 1965) was used to assess respondents’ level of self-esteem. Adolescents filled in a Polish version of the scale. This scale consists of ten items rated on a 4-point scale (from “1 = strongly disagree” to “4 = strongly agree”). Higher scores indicate better self-esteem. RSES has been extensively used among Polish adolescents, showing adequate psychometric properties (e.g., Delvecchio, Lis, Liberska, & Li, 2017; Dzwonkowska, Lachowicz-Tabaczek, & Łaguna, 2008). Sample items include “I feel that I have a number of good qualities”. The Cronbach’s α was .82 in the current study.

### Procedure

This study was conducted in compliance with the ethical standards for research outlined in the *Ethical Principles of Psychologists and Code of Conduct* (American Psychological Association, 2010). Approval by the Ethical Committee for Psychological Research was obtained from university of Bydgoszcz. School approval and parents’ signed consent were sequentially obtained before data collection and adolescents provided their assent before participation. No incentives were awarded; voluntary participation and anonymity were emphasized. Participants completed the questionnaires during regularly scheduled classes and were instructed to be open and honest in their responses and to refrain from sharing answers with each other. Administration was conducted in compliance with the standard procedures.

### Data Analyses

Multiple analyses were carried out using SPSS 18.0. First, descriptive statistics (means and standard deviation) were calculated. Second, independent *t*-test was carried out to examine whether there would be gender differences in the total and dimension scores. Substantial difference was determined when *t* value was significant (i.e., *p* < .05) and effect size (i.e., Cohen’s *d*; Cohen, 1992) was at least medium. Third, paired *t*-test was performed to investigate the preferred attachment figure. Fourth, correlations between the total and dimension scores of attachment and self-esteem were tested for the overall sample and separately for boys and girls. Effect sizes of correlation coefficient were checked, with .10, .30, and .50 as small, medium, and large effect size, respectively (Cohen, 1992). In order to investigate whether the correlations differed between boys and girls, Steiger’s *Z* test (Steiger, 1980) was conducted. Finally, Polish adolescents’ attachment total and dimension scores were compared with previous results obtained from Chinese and Italian samples (Li et al., 2014) using one-sample *t*-test.

## Results

### Description of and Gender Differences in Parental Attachment

Total and dimension scores of maternal and paternal attachment in the overall sample and for boys and girls as well as the results of independent *t*-test by gender are presented in Table 1. No significant gender difference in total and dimension scores of maternal and paternal attachment was found, suggesting that levels of parental attachment were similar for boys and girls.

**Table 1:** Descriptive Statistics of and Gender Differences in Parental Attachment

IPPA-R scale	*M* ± *SD*			*t*(301)	*p*	Cohen’s *d*
	Overall	Boys	Girls			
Maternal attachment	91.64 ± 18.69	92.39 ± 19.54	90.60 ± 17.47	0.821	.412	0.095
Maternal Trust	38.35 ± 8.01	38.68 ± 8.06	37.90 ± 7.95	0.841	.401	0.097
Maternal Communication	31.81 ± 7.19	32.14 ± 7.63	31.36 ± 6.53	0.925	.356	0.107
Maternal Alienation	14.53 ± 5.23	14.43 ± 5.49	14.66 ± 4.86	-0.377	.707	0.043
Paternal attachment	85.33 ± 18.90	83.88 ± 19.41	87.35 ± 18.05	-1.579	.115	0.182
Paternal Trust	36.08 ± 8.35	35.47 ± 8.51	36.94 ± 8.07	-1.517	.130	0.175
Paternal Communication	28.90 ± 7.62	28.21 ± 7.94	29.86 ± 7.08	-1.864	.063	0.215
Paternal Alienation	15.65 ± 5.07	15.80 ± 4.88	15.45 ± 5.34	0.587	.558	0.068

### Preferred Attachment Figure

Paired *t*-test was conducted to examine preferred attachment figure. The results showed that participants reported higher levels of total attachment to mother than to father, *t*(302) = 4.834, *p* < .001, higher trust to mother than to father, *t*(302) = 4.235, *p* < .001, higher level of communication with mother than with father, *t*(302) = 5.454, *p* < .001 and less alienation from mother than from father, *t*(302) = -3.334, *p* = .001.

### Association With Self-Esteem

As shown in Table 2, maternal and paternal total attachment and dimension scores were significantly associated with self-esteem for the overall sample, but some differences in effect size were found. To be more specific, maternal total attachment and dimension scores were correlated with the RSES at medium effect sizes whereas paternal total attachment and dimension scores were correlated with RSES at low effect sizes. Moreover, significant differences in the correlation coefficients between boys and girls occurred. First, there was no significant difference between boys and girls in the relationship between maternal attachment and self-esteem except for maternal communication, as evidenced by the results of Steiger’s *Z* test shown in Table 2. The relationship between communication with mother and self-esteem was significantly higher for boys than for girls.

**Table 2:** Correlations Between Parental Attachment and Self-Esteem for the Total Sample and Divided by Gender

IPPA-R scale	Total sample	Boys	Girls	*Z*	*p*_two-tailed_
Maternal attachment	.45**	.50**	.40**	1.07	.285
Maternal trust	.41**	.42**	.41**	0.10	.920
Maternal communication	.38**	.46**	.26**	1.96	.050
Maternal alienation	-.46**	-.51**	-.40**	-1.18	.238
Paternal attachment	.26**	.11	.47**	-3.40	< .001
Paternal trust	.26**	.12	.45**	-3.09	.002
Paternal communication	.18**	.10	.28**	-1.59	.112
Paternal alienation	-.28**	-.09	-.52**	4.13	< .001

*Note.* Steiger’s Z test for gender differences evaluation.

**p < .01.

A totally different picture appeared for paternal attachment. There were no significant relationships between paternal attachment and self-esteem among boys. However, there were differences in girls. Correlations for girls were at medium level with the exception of communication as shown in Table 2. Results of of Steiger’s *Z* test confirm this significant difference.

### Comparison With Previous Studies

One-sample t-tests were carried out to compare the current results with previous research done among Chinese and Italian samples (Li et al., 2014) since the age groups of that research was comparable to the current one. Because factor scores were reported in Li et al.’s (2014) paper, the first author of that study was contacted for the raw data for comparison.

As shown in Table 3, some significant differences between Polish and Chinese adolescents were found. Polish adolescents reported higher level of maternal total attachment, trust and communication than their Chinese counterparts. As for fathers, although no significant differences were found for total attachment, Polish adolescents reported lower level of trust, higher level of alienation, but higher level of communication than their Chinese counterparts. Regarding the comparison between Polish and Italian adolescents, except the case of paternal communication, Polish youth in general reported lower level of attachment to both father and mother (as indicated by lower total attachment score, less trust, communication, and more alienation), than did Italian adolescents.

**Table 3:** Comparison With Previous Chinese and Italian Data

IPPA-R scale	*M* ± *SD*			*t*_Poland-China_	*p*	*t*_Poland-Italy_	*p*
	Poland (*n* = 303)	China (*n* = 350)	Italy (*n* = 352)				
Maternal attachment	91.64 ± 18.69	87.54 ± 15.37	95.13 ± 16.70	3.816	< .001	-3.253	.001
Maternal Trust	38.35 ± 8.00	37.18 ± 6.80	41.55 ± 7.44	2.551	.011	-6.950	< .001
Maternal Communication	31.81 ± 7.19	29.18 ± 6.76	33.14 ± 7.40	6.372	< .001	-3.215	.001
Maternal Alienation	14.53 ± 5.23	14.82 ± 4.30	12.85 ± 4.80	-.972	.332	5.587	< .001
Paternal attachment	85.33 ± 18.90	86.07 ± 16.05	87.61 ± 17.58	-.679	.498	-2.097	.037
Paternal Trust	36.08 ± 8.35	37.41 ± 7.18	39.50 ± 7.89	-2.769	.006	-7.128	< .001
Paternal Communication	28.90 ± 7.62	27.54 ± 7.09	28.46 ± 7.89	3.108	.002	1.007	.315
Paternal Alienation	15.65 ± 5.07	14.88 ± 4.88	13.82 ± 4.75	2.644	.009	6.284	< .001

## Discussion

The first aim of this study was to assess levels of attachment as well as gender differences and preferred attachment figure among Polish adolescents using the IPPA-R. In line with previous studies, the IPPA-R appeared to be a reliable tool, with high levels of internal consistency for the total and dimension scores (Farnicka & Grzegorzewska, 2015; Li et al., 2014; Pace, San Martini, & Zavattini, 2011). No significant difference was found for gender for maternal or paternal total attachment and dimension scores. These findings confirmed previous studies in IPPA-R with adolescents (Li et al., 2014). However, they can be also explained referring to the social Polish context: commitment to the family and relatives, longing for the warm from the family circle, and the sense of belonging to a family are still strong in the younger generation, in spite of their gender (Dyczewski & Jedynak, 2002) and this may be a possible reason why there was no significant difference in total attachment, trust, communication and alienation between boys and girls.

Polish adolescents reported significantly stronger attachment to mothers than to fathers, showed to trust and communicate significantly more with mothers than fathers and to feel less alienated toward mothers than toward fathers. These findings can be interpreted in view of Polish sociopolitical and culture context. Under the communist revolution women behaved practically like the sole care-givers of children, taking care of all of childcare duties and being responsible for child wellbeing. Fathers, although considered the authorities within families, were uninvolved in child rearing. Poland was seen as a patriarchal country in which children were expected to develop strong emotional ties with mothers and less affectionate relationships with fathers. Skorupska-Sobańska (1971), empirically confirms this tendency showing that most of children believed that mothers would support them during times of difficulty. Only a paucity of them (3%) had the same thought referring to fathers’ availability. Polish teenagers perceived mothers as caring, devoted to children, interested in their difficulties as well as in daily life issues, while fathers were perceived as controlling, authoritative, reluctant to spend time together and not interested in their lives. With the collapse of communism, trends toward democratization have started to influence parenting roles by overcoming cultural traditionalism. Fathers became more able to cross parental role boundaries helping with childcare and domestic duties more extensively (Wejnert & Djumabaeva, 2005). Nonetheless, in this study Polish adolescents reported significantly stronger attachment to mothers than to fathers.

Findings about relationship between parental attachment security and self-esteem seemed to confirm the importance of maternal versus paternal attachment in Polish adolescents’ development of positive self-evaluation. Specifically, maternal attachment helps Polish adolescents, both boys and girls, to feel the self as competent. However, stronger paternal attachment provides some contribution to girls’, rather than boys’, perception of themselves as positive and competent. Maybe boys, more than girls, need a progressive improved father-child relationship in order to improve their self-esteem. Wejnert and Djumabaeva's (2005) suggested that the encouragement of Western examples of parenting should progressively help improve father-child relationships in Poland and that the models of involved and active parenting are especially important within liberal democratic systems where the parent-child relationships are challenged by increases in youthful drug use, teen prostitution, as well as teen and out-of-wedlock pregnancy (Wejnert & Djumabaeva, 2005).

Another important aim of this paper was to compare Polish adolescents’ quality of attachment with the findings obtained from collectivistic non-Western society (China) and with the ones from individualistic Western society (Italy). The results showed that levels of parental attachment among Polish adolescents were generally higher than Chinese but lower than Italian counterparts. Regarding Polish adolescents, although the sense of belonging to a family is considered still strong in the younger generation and young people in Poland remain closely to their families longer and more comprehensively than in other Western countries (Dyczewski & Jedynak, 2002), their attachment tie was not so strong as compared with Italian adolescents. It is important to take into account that the previous Polish generation was embedded in a collectivistic orientation where the primary values were at a social level and so may be not characterized by a high degree of emotional bonding as in Italy. Even though the younger generation remains closely connected, they do not look like to have a strong emotional bond with their parents as compared with Italian adolescents; notwithstanding under the recent individualistic orientation a greater sense of independence is now present against these ties. Also as observed by Hardt et al. (2011) it may reflect the wish for mothers that their child develop well socially and attain a better (economic) status than the family have, more than to commit themselves to an affectionate bond so the mother continues to maintain the task of managing the family in Poland but with different aims than attachment. Moreover, may be, mothers’ experience of their personal life after the collapse of communism could have led them to be more preoccupied by off-family tasks related with macro-processes linked with transformation, and for these reasons less concerned about parenting or child-care. In conclusion, adolescents’ attachment in Poland as compared with other collectivistic as well individualistic country seems to be connected with its history and sociopolitical changes.

The present study is not without limitations. The Polish sample was only drawn from one economically and educationally developed metropolitan, and thus the generalizability of the results to overall sample is limited. Moreover, only self-report questionnaires were used. Third, the current research adopted a cross-sectional approach. Future longitudinal studies need to track the psychological and social changes occurring in the Polish. Nevertheless, the current study contributes to literature by revealing level of parental attachment in Poland in the context of cultural changes. Moreover, this research also adds to the limited cross-cultural literature of parental attachment. In conclusion, we hope these findings can provide an initial view of Polish adolescents’ attachment to parents in the ongoing cultural context and lay the ground for future research.
